# Spine Cop: Posture Correction Monitor and Assistant

**DOI:** 10.3390/s20185376

**Published:** 2020-09-19

**Authors:** Pedro Ribeiro, Ana Rita Soares, Rafael Girão, Miguel Neto, Susana Cardoso

**Affiliations:** 1 Instituto de Engenharia de Sistemas e Computadores—Microsystems and Nanotechnologies, 1000-019 Lisbon, Portugal; asoares@inesc-mn.pt (A.R.S.); rgsantos@criticalsoftware.com (R.G.); mneto@inesc-mn.pt (M.N.); scardoso@inesc-mn.pt (S.C.); 2 Instituto Superior Técnico, Universidade de Lisboa, 1049-001 Lisbon, Portugal; 3 Critical Software, 3045-504 Taveiro, Portugal

**Keywords:** ergonomics, wearable device, posture, accelerometer, magnetometer

## Abstract

Back and spine-related issues are frequent maladies that most people have or will experience during their lifetime. A common and sensible observation that can be made is regarding the posture of an individual. We present a new approach that combines accelerometer, gyroscope, and magnetometer sensor data in combination with permanent magnets assembled as a wearable device capable of real-time spine posture monitoring. An independent calibration of the device is required for each user. The sensor data is processed by a probabilistic classification algorithm that compares the real-time data with the calibration result, verifying whether the data point lies within regions of confidence defined by a computed threshold. An incorrect posture classification is considered if both accelerometer and magnetometer classify the posture as incorrect. A pilot trial was performed in a single adult test subject. The combination of the magnets and magnetometer greatly improved the posture classification accuracy (89%) over the accuracy obtained when only accelerometer data were used (47%). The validation of this method was based on image analysis.

## 1. Introduction

Different kinds of pain and dysfunction are frequent maladies that most people have or will experience during a lifetime, which are usually related to sports injuries, an accident, or other medical conditions, such as scoliosis. However, most of the upper or lower back pains are developed during the repetitive activities of day-to-day life. According to the Harvard Medical School, an efficient strategy to prevent them is as simple as paying attention to your posture [1,2]. Measurement of human posture and movement is an important area of research in the bioengineering and rehabilitation fields. However, posture is not an easy subject to study, quantitatively or qualitatively. Currently, there is a wide range of tools to measure posture; however, there is no gold standard method to measure posture. Various attempts have been initiated for different clinical application goals, such as diagnosis of pathological posture and movements combined with the assessment of pre- and post-treatment efficacy.

There is a wide range of posture and back shape assessment tools available, some of which are for clinical use, such as photography, goniometers, inclinometers, 2D and 3D X-ray imaging, magnetic resonance imaging, and infrared imaging. However, most of the assessments are still expensive, complicated to set up, time-consuming to operate, and need specialized training or involve radiation problems [3].

The radiographic methods are the most used tools for the screening of the spines of patients; notably, 3D X-ray imaging produces an accurate 3D reconstruction of anatomical landmarks per vertebra. On the other hand, these tools have been shown to increase the incidence of cancer in later years, are complex to set up, are heavy, and can only be applied in laboratory environments [4]. Moreover, most of them could only be applied in laboratory environments, which means that the subjects are aware that their postures are being analyzed and testing cannot take place in usual environments. Most commonly, in both research and clinical settings, cervical posture is measured in two dimensions with sagittal plane views of head or neck posture [5]. A sensible observation that can be made is regarding the posture of an individual. Maintaining a good posture throughout the different activities of the day is an important way for preserving spinal health. Furthermore, corrective exercises can be employed in the treatment of some back issues, namely scoliosis [6]. With this in mind, posture monitoring can be either a useful mechanism to assist in therapy or a day-to-day tool for an aware user to correct and adapt their posture in different situations such as sitting down during work, carrying weights, etc.

There are several solutions involving the measurement of the inclination and symmetry about the body sagittal plane [7]. These devices employ flexible inductive sensing [8,9] or flexible piezoelectric sensors [10,11], inertial measurement unit (IMU) devices that contain accelerometer, gyroscope, and magnetometer sensors [4,12,13]. These portable and wearable tools are inexpensive and user-friendly, and they have been demonstrated to be suitable for posture monitorization. However, there is a lack of information about the reliability of these tools for measuring spinal curvatures or other back parameters [3]. The limitations of these tools are related with the fact that these devices only monitor specific regions of the back and do not provide information about the whole-back surface.

Photographs can also quantify postural assessment by measuring the body angle or distance to globally assess posture both in sagittal and frontal planes, and the acquisition is cheap, fast, and easy [5,14]. Some researchers and experienced clinical users reached out to say that these sensors individually can be insufficient to perform a clinically relevant test and suggested the support of the different methods to improve the accuracy of the measurements [4,11].

The Spine Cop is a wearable posture correction and monitoring assistant system based on the SensorTile compact module [15], a Bluetooth Smart board that combines several sensors modules such as an LSM6DSM–iNEMO inertial module [16] (3D accelerometer and 3D gyroscope) and Ultra-compact high-performance LSM303AGR eCompass module [17] (3D accelerometer and 3D magnetometer), among other sensors that are not used in this work. As a new approach, explicitly using the raw magnetometer data, permanent magnets were integrated with the SensorTile, generating a magnetic signal that can be used to describe the movement of the shoulder blades, and is captured by the SensorTile magnetometer, showing an improvement in the postural classification accuracy of the device. With these elements attached to moveable pieces of cloth, the ability to distinguish if its user maintains a correct posture is possible, even when the user performs actions or poses that could interfere with the postural assessment. Using an improved sensing scheme, this device is set to achieve a portable, non-intrusive, comfortable wearable device that is capable of real-time monitoring for long periods, either in a medically supervised postural monitorization (therapeutic use) or for personal use, which means without medical assistance. SensorTile is expected to bring benefits in terms of both ease-of-use and device autonomy due to its native Bluetooth connectivity and low power consumption within a small and easy-to-integrate package.

## 2. Materials and Methods

**Figure 1:**
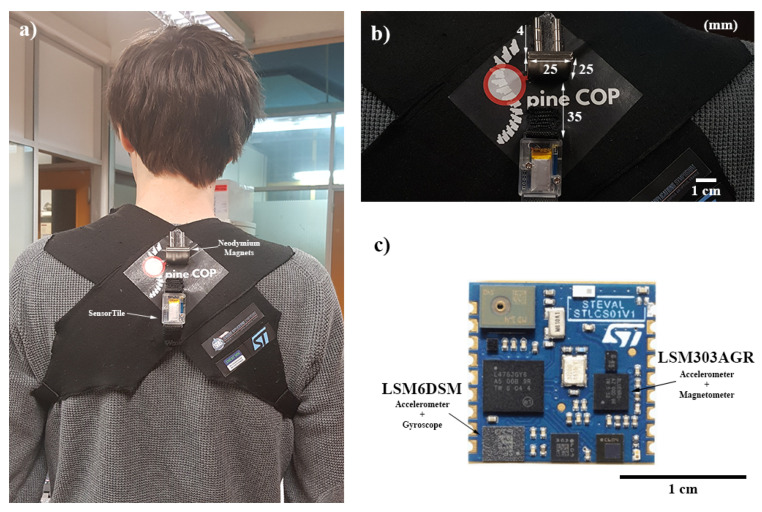
(**a**) User wearing the SpineCop wearable device. (**b**) Detailed view of the SpineCop device. (**c**) SensorTile PCB. The used sensors (accelerometer, magnetometer, and gyroscope are indicated).

This wearable device consists of a suspensory with the SensorTile located between the shoulder blades and two magnets in the region above (Figure 1).

The area where the rigid components are placed corresponds to the areas near the cervical curve (slight forward curve in the neck)—permanent magnet position—and the thoracic curve (slight backward curve in the upper back)—SensorTile module position—since these are two points used for postural assessment [18].

**Figure 2:**
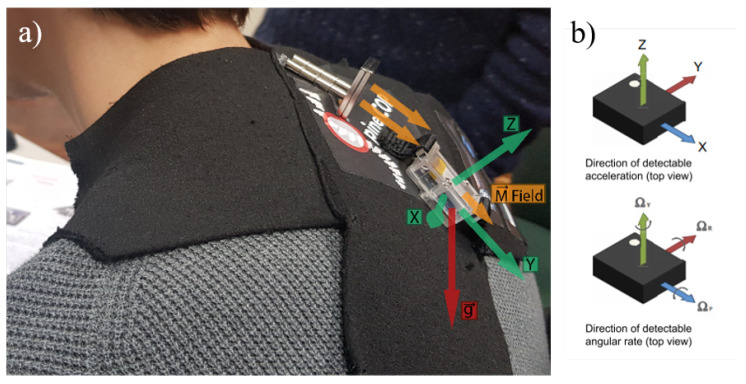
(**a**) Magnetic field and accelerometer orientations of the SensorTile device on the wearer’s back. (**b**) Accelerometer and gyroscope orientations on the SensorTile device (LSM6DSM chip).

Two posture parameters can be analysed: the movement of the shoulder blades, which will induce a change in the position of the permanent magnets (cervical curve) that will cause a magnetic signal variation in the magnetometer sensor placed in the thoracic curve (referential); and the angle that the thoracic tangent line makes with Earth’s gravitational field (Figure 2), using only one SensorTile module.

The monitoring of the shoulder blades’ muscles is accomplished by the magnetometer since the function of these muscles will affect the position of both N45 type magnets [19]. We viewed the ideal skeletal alignment as one in which the line of gravity, when viewed from either the front or the back, should theoretically bisect the body into two halves, with the bodyweight distributed evenly between the two feet. Thus, when the shoulders are on the anatomically ideal position, the magnets should be closer to the SensorTile, while the opposite happens as the posture worsens. According to the magnetometer sensitivity and considering the magnetic field dynamic range of ±5 mT [17], the magnets integrated into the system guarantee a signal strong enough, 2.2 mT, to achieve the required resolution to classify the shoulder blade positions.

The accelerometer consists of a three-axis microelectromechanical system (MEMS) accelerometer that measures the acceleration along the three sensitive axes of the sensor reaching the spine relative angle with the line of gravity in up to three planes, in up to ±400 g acceleration full scale [16,17]. In a static measurement, they measure the tilting angle concerning the axis of the gravitational field.

The gyroscope is a sensor used to measure angular velocity and, jointly with the accelerometer (for orientation estimation in 3D space), is used to provide information on the current stance of the user (i.e., just standing or walking/running). A small dimension lithium-ion battery with 100 mAh of capacity is used to ensure the comfort of the user.

The posture scoring will be calculated based on experimentally obtained thresholds for the magnetometer and accelerometer values. The integrated microcontroller will be monitoring the IMU (inertial measurement unit) values in real time, sending the data to the application for posture processing. For each data point transmitted to the application, a binary classification (good or bad posture) will be attributed. At the end of a Spine Cop session, a global posture score will be computed based on all individual scores.

## 3. Algorithm

A probabilistic algorithm was implemented to classify the user posture. A calibration procedure is performed first, during which the user is asked to perform a correct posture, which is registered. Afterwards, the device data are compared with the calibration result, and the posture classification is performed.

As the user can only be asked to perform a good posture for calibration, the determination of an optimal threshold for good and bad posture is not possible. Therefore, the thresholds for determining the regions where accelerometer and magnetometer data indicate good and bad posture are statistically estimated.

This estimation is done by acquiring a set of values while the user is performing different poses. These sets of values are then fitted to a Gaussian distribution, returning a mean vector and a covariance matrix, which can then be used to define an arbitrary region of confidence (95% was chosen for this case), within which the posture is considered to be good. When the user posture wanders away from these regions (i.e., effectively deviating his posture from the calibrated one, with an uncertainty of less than 5%), the system classifies the user posture as incorrect.

An independent calibration procedure is required for each individual user; however, the method for calculating these thresholds is the same for all users.

### 3.1. Calibration and Thresholds

Magnetometer and accelerometer data were extracted from the SensorTile system to assess, through the classification algorithm, the posture of the user. Both of these sensors provide a three-element vector with values corresponding to the acceleration and magnetic field in the three spatial components, respectively.

During the calibration procedure, the user is asked to assume a set of positions *i* while keeping a good posture, and the values measured for each position are recorded into a set of values $S_{i}$.

Good posture is defined as the correct skeletal alignment of the cervical, thoracic, and lumbar curves and musculoskeletal balance. The test subject was asked, during the calibration procedure, to assume a good posture, requiring the ear, shoulder top, hip, knee, and the ankle to line up vertically on standing stance, when seen from the side [1].

For this work, eight calibration positions were chosen, with user keeping a straight back while having:

- Both arms down
- Right arm up
- Left arm up
- Both arms up

**Figure 3:**
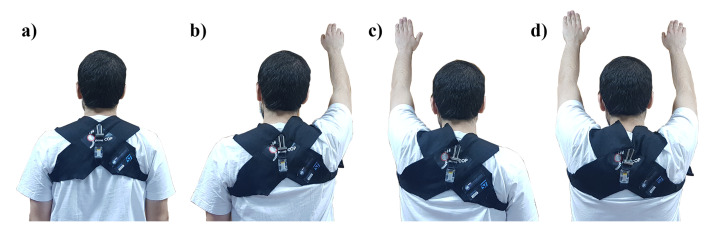
Various calibration positions imposed to the user. (**a**) Both arms down. (**b**) Right arm up. (**c**) Left arm up. (**d**) Both arms up.

These different arm positions allow the classification algorithm to account for arm and hand movement during the usage of the device, thus allowing it to perform a correct posture classification even while the user is doing arm and hand movements (e.g., while working). The device was calibrated while the user was standing up (four calibration positions) and sitting down (four calibration positions). A photo of the various calibration positions is presented in Figure 3.

The set of values $S_{i}$ for each sensor are then each fitted to a trivariate Gaussian distribution,

(1) $$N_{i}\left(x,\mu_{i},\Sigma_{i}\right)={\left(2\pi \right)}^{-\frac{3}{2}}{\left|\Sigma_{i}\right|}^{-\frac{1}{2}}\;e^{-\frac{1}{2}{\left(x-\mu_{i}\right)}^{\;T}{\Sigma_{i}}^{-1}\left(x-\mu_{i}\right)},$$

where $\mu_{i}$ is the mean vector of the *i* dataset estimated by (with *k* representing one of the acquired values in the set)

(2) $$\mu_{i}=\frac{1}{N}\sum_{k=1}^{N}S_{ik},$$

and $\Sigma_{i}$ is the covariance matrix of dataset $S_{i}$ estimated by

(3) $$\Sigma_{i}=\frac{1}{N-1}\sum_{k=1}^{N}\left(S_{ik}-\mu_{i}\right){\left(S_{ik}-\mu_{i}\right)}^{T}.$$

The device was designed with the intention of being capable to be used by any adult male or female. However, different people will exhibit different body behaviors, leading to different thresholding limits. Therefore, it is of interest that the posture thresholds can be computed in a singular manner using the data acquired during calibration.

The acquired data of the sensors are expressed in the real-world spatial components *X*, *Y*, and *Z*. These data are fitted to a Gaussian distribution, which will present equiprobability surfaces that can be described by ellipsoids in a three-dimensional space. However, the signal in one spatial component is co-dependent on the signal measured in the remaining components (i.e., its axes are not aligned with the spatial system of coordinates axes). Transforming the system of coordinates into the principal components of the data (*x*, *y*, and *z*), this ellipse can be more easily expressed. Thus, the trivariate Gaussian distribution presents, in the principal component space, an elliptical shape that can be expressed as (using the signal from a sensor $S_{i}$)

(4) $${\left(\frac{S_{ix}}{\sigma_{ix}}\right)}^{2}+{\left(\frac{S_{iy}}{\sigma_{iy}}\right)}^{2}+{\left(\frac{S_{iz}}{\sigma_{iz}}\right)}^{2}=s$$

where $\sigma_{j}$ represents the standard deviation of the signal along a dimension *j*, and *s* defines the scale of the equiprobability ellipsoid. On a multivariate Gaussian distribution, *s* is $\chi_{k}^{2}$ (chi-squared) distributed (with the *k* degrees of freedom corresponding to the number of dimensions of the distribution—in this case, k=3).

Assuming an arbitrarily defined interval of confidence *c* is chosen for the trivariate Gaussian distribution, then $s=\chi_{3}^{2}\left(c\right)$. From Equation (4), it can be derived that the distance between the probability corresponding to the limit of the confidence interval *c* and the mean vector $\mu_{i}$ (i.e., half the size of one of the Gaussian ellipses axes) is

(5) $$\delta_{j}=\sqrt{\chi_{3}^{2}\left(c\right)\lambda_{j}}\;v_{j}$$

where $\lambda_{j}$ and $v_{j}$ are the eigenvalue and eigenvector of $\Sigma_{i}$ corresponding to dimension *j*. Finally, the threshold probability τ for a calibration set *i* can be computed as (from Equation (1))

(6) $$\tau_{i}=N_{i}\left(\mu_{i}+\delta_{i},\mu_{i},\Sigma_{i}\right).$$

For this work, the *c* used in the calibration procedure is 0.95.

### 3.2. Posture Monitoring and Thresholds

**Figure 4:**
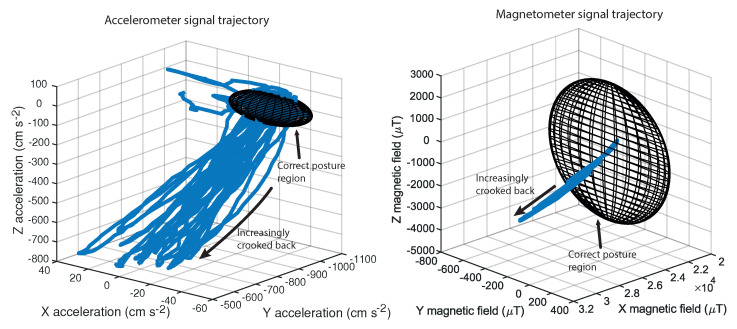
Regions of confidence for a correct posture while standing with arms down (meshed ellipsoid) and trajectory of sensor data during a test (blue line). The plot on the left is related to accelerometer data, while the plot on the right is related to magnetometer data.

The posture monitoring is based on the implemented probabilistic algorithm. When the algorithm receives a data point relative to one of the sensors, the classification of correct or incorrect posture is performed by verifying whether the data point lies within the regions of confidence defined by the computed thresholds τ of the fitted Gaussians N for all the *i* calibration sets (Figure 4).

It is also interesting to note from Figure 4 that the magnetic field changes in 1 mT when the back shifts from a correct posture to a wrong one. This is much higher than the manufacturer reported 3 μT sensitivity.

The posture classification is made based on the individual result provided by the accelerometer and magnetometer classifiers. If the accelerometer or magnetometer classifier determines that the posture is correct, the posture is classified as correct. For the posture to be classified as incorrect, both the accelerometer and magnetometer classifiers must classify the posture as incorrect (Table 1).

### 3.3. Spine Cop Score

Although real-time posture monitorization is of interest for the user, it is also of interest to have a figure of merit that the user can take advantage to assess the performance throughout a long period of time, as well as to track his posture improvement in the following days.

**Figure 5:**
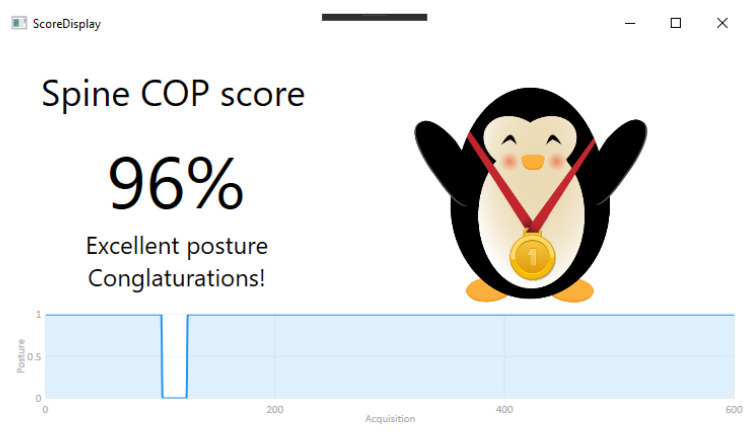
Posture classification over a 2 min period. In this case, the user performed the correct posture for 96% of the test duration.

The Spine Cop score is a tool that saves the posture classification after an arbitrary period of time (up to 24 h) and returns a score of the user posture. The score is given as the percentage of that time interval the user kept a correct posture (Figure 5).

### 3.4. Straigth Back with Tilting Test

The usage of the magnet/magnetometer combination is expected to increase the presented device classification performance, especially in cases in which the back is not perpendicular to the ground, but is still kept straight.

For this test, we test one particular case, in which the user tilts his back while keeping it straight (keeping a correct posture through the entirety of the test). It would then be expected, ideally, that the posture would be classified as correct for the whole duration of the test.

If only the accelerometer was used, it would be expected that the posture would be wrongly classified during the test, as it only compares the back angle with the gravitic earth field.

### 3.5. Crooked Back Test

In the crooked back test, the user was asked to repeatedly alternate between having his back in a straight and crooked position, while the computed posture state was monitored.

### 3.6. Back Angle Validation

The angle of the user’s back $\alpha_{\mathrm{ST}}$ is calculated by computing the angle between the acceleration vector measured by the SensorTile device $a_{\mathrm{ST}}$ and the gravitational acceleration vector g, using:

(7) $$\alpha_{\mathrm{ST}}=\arccos\left(\frac{a_{\mathrm{ST}}\cdot g}{|a_{\mathrm{ST}}\left||g\right|}\right).$$

**Figure 6:**
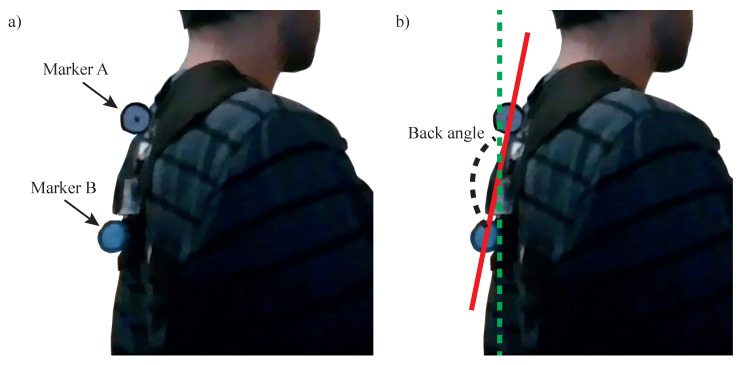
(**a**) Photograph of the user wearing the device. The two round markers were used to allow the Kinovea software to track the movement of the back during the exercise. (**b**) Used back angle. The angle of the back is taken as the angle between the line that crosses both markers and a vertical line (parallel to the gravitational acceleration vector).

To validate the accuracy of this measurement, the angle of the back was also measured through image analysis. The device was fitted with two markers (*A* and *B*) and a video was recorded of the user bending his back into a correct and incorrect posture, while the position of the markers was recorded using the Kinovea image analysis software (Figure 6a). The angle between the straight line crossing these markers and a vertical line (gravitational acceleration vector) was used to calculate the angle through the video (Figure 6b).

**Figure 7:**
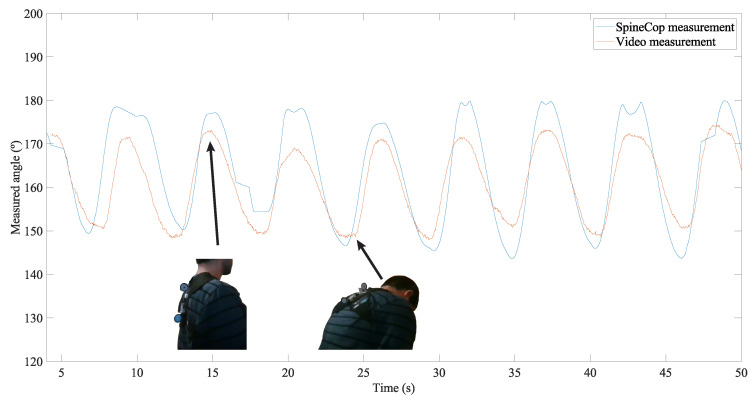
Angles measured by the SensorTile (blue line) and the video analysis (orange line). The positions performed by the user are shown in the plot.

The measurement was made while the posture was alternated 10 times, and the results for both measurement methods are presented in Figure 7.

An agreement can be observed between both types of measurements, with a RMS difference of 5${}^{\circ }$.

## 4. Results

The classifier performance was tested for a single test subject and assessed in terms of accuracy, defined as the percentage of time it provided a correct posture classification. The subject consent was given to perform the experiment, with knowledge of the final purpose of the acquired data. Furthermore, the test subject identity was not recorded.

### 4.1. Straight Back with Tilting

However, with the added magnets placed in the back together with the SensorTile (with an integrated magnetometer), an estimation of the back actual angle can be performed, thus allowing the classification to be performed correctly within a greater variety of positions.

A single test was run, and the classification was run using only accelerometer data, and both accelerometer and magnetometer data, thus allowing the assessment of how much the additional magnetometer data can improve the performance of our classification algorithm (and, more generally, of the device).

**Figure 8:**
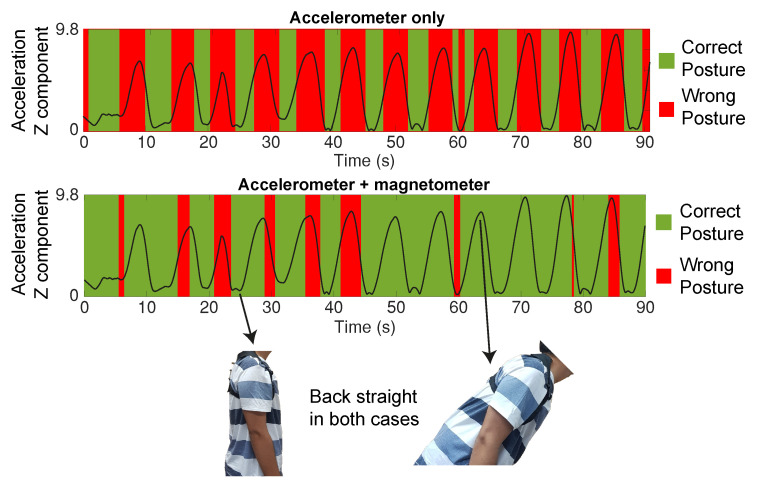
Posture quality classification result using accelerometer data only, accelerometer and magnetometer data simultaneously, and the posture assumed by the user during the test. The black line represents the measured acceleration in the Z component by the sensor. Despite the user tilting his back, it is kept straight during the entirety of the test.

The classification algorithm results for both devices are shown in Figure 8.

It was observed that when the classification algorithm used both accelerometer and magnetometer data, it achieved an accuracy of 85%, much higher than the case where only accelerometer data is used, with 47% accuracy.

This confirms that the usage of both values greatly improves the device performance over the usage of accelerometer data only, especially when the user performs movements or poses that are different from just normal standing or sitting (i.e., with the back perpendicular to the ground).

### 4.2. Crooked Back

Both the magnetometer and accelerometer data were used in the classification. For the purpose of validation, we considered the user to have a posture different than the calibrated one when the back angle (calculated using accelerometer data) falls below 160${}^{\circ }$. From the video analysis, we have a high degree of confidence that a back angle below 160${}^{\circ }$ corresponds to a back posture substantially different from the calibrated one. This can provide an upper limit of accuracy that will most likely be lower should an anatomically correct testing be performed.

**Figure 9:**
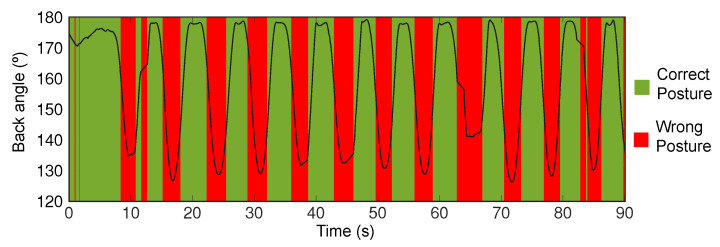
Posture classification using algorithm (green and red bars) and measured back angle (black line).

The expected and obtained classifications results agree with each other (Figure 9) and yield a classification accuracy of 89% for this test.

## 5. Discussion

The device presented in this paper is not directly comparable with the other devices reported in the literature, as our device employs a probabilistic method, and other devices have either not considered the correctness of the measured posture [8] or directly measure angles in certain points of the lumbar region [13].

The main advantage of our device is in its compactness, requiring just contact with two points of the back when compared with other devices which require carrying large and heavy data conversion and wireless modules [8], and the usage of large sensing elements [13].

Furthermore, the usage of the magnet to provide information about the posture of the cervical portion of the spine provides a simplification in the device design, leading to lower power consumption and production cost.

The performed study has potential limitations, specifically in what concerns the number of tested subjects and calibration of the device with a correct posture.

Regarding the first point, only one test subject was thoroughly tested by the presented studies above. As is, there are not enough data to statistically confirm that the device functions correctly for the whole demographic for whom it has been designed (adults).

As for the second point, the standard for correct posture was taken from literature [1] and the test subject was asked by the authors of this article (who are not medical experts) to assume the reported posture. Furthermore, the validation of the device was made by comparing the posture result with the angle of the back, calculated using a recorded video of the test subject performing the test tasks, a relatively low fidelity parameter to which the sensor is compared. Therefore, we believe the accuracy assessment in this work to be increased over the actual one. Should the posture and validation process be performed by a medical professional, we would expect the classification accuracy to be inferior to the one reported on this article.

To overcome these limitations, a greater test subject ensemble should be recruited and a statistical study conducted. To obtain an anatomically accurate assessment of the device performance, future tests should be conducted by a medical professional, specifically one with knowledge of orthopedics or ergonomics (or a combination of both).

## 6. Conclusions

We developed a wearable posture monitoring system based on the SensorTile module using a combination of sensor data (accelerometer, magnetometer, and gyroscope) with permanent magnets to assess the user’s back position.

Through a statistical algorithm, the classification of the posture was performed and validated.

The usage of magnetometer data in combination with accelerometer data displayed an improved performance (85% accuracy) when compared with using the accelerometer data alone (47%). Moreover, in a test where the user alternated between a good back position and a crooked one, an accuracy of 89% was obtained.

For future work, a more thorough validation process, with posture positions anatomically verified by a physician will be performed. Furthermore, a real-time monitoring for a day-long period of time is also planned.

## Figures and Tables

**Table 1:** Posture classification truth table

Accelerometer-Based Posture Classification	Magnetometer-Based Posture Classification	Final Posture Classification
Incorrect	Incorrect	Incorrect
Incorrect	Correct	Correct
Correct	Incorrect	Correct
Correct	Correct	Correct
